# Clinical Features and T Cell Immune Characteristics of Postpartum Hepatitis Flare in Pregnant Women With HBeAg-Positive Chronic HBV Infection

**DOI:** 10.3389/fimmu.2022.881321

**Published:** 2022-04-14

**Authors:** Aixin Song, Yisi Liu, Zhenhuan Cao, Junfeng Lu, Shan Ren, Sujun Zheng, Lina Ma, Zhongjie Hu, Xiao Lin, Hong Li, Yanhong Zheng, Xinyue Chen

**Affiliations:** First Department of Liver Disease Center, Beijing Youan Hospital, Capital Medical University, Beijing, China

**Keywords:** HBV, postpartum, alanine transaminase, hepatitis flare, CD8^+^ T cells

## Abstract

**Background:**

The extent of the increase in postpartum alanine transaminase (ALT) varies significantly among pregnant women in the immune tolerance stage of nucleoside analogue (NA) intervention, so this study is an attempt to analyze the clinical features of patients with and without postpartum hepatitis flare and preliminarily explore the differences in their immune functions.

**Methods:**

Pregnant women with a gestational age of 24–28 w and in the immune tolerance stage of NA intervention for hepatitis B virus (HBV) infection were included and divided into a hepatitis group (Group 1) and a nonhepatitis group (Group 2) according to the ALT level at 6–12 w after childbirth. The clinical features were analyzed, and the phenotypes, functions, and cytokines of clusters of differentiation CD8^+^ T cells in the two groups of patients were detected using flow cytometry before and after childbirth.

**Results:**

A total of 15 patients with postpartum hepatitis flare were enrolled in Group 1, and 10 matched patients were selected as controls for Group 2. Compared with the individuals in Group 2, the postpartum clinical features in Group 1 included a remarkable elevation of the ALT level on the basis of a relatively low HBV DNA level, usually accompanied by a decline in hepatitis B virus surface antigen levels as well as HBeAg levels. In addition, CD8^+^ T cell activation was enhanced after childbirth in Group 1. In particular, there was a notable difference in the activation of TEMRA subsets, and the frequency of CD8^+^ T cells expressing perforin and granzyme B increased.

**Conclusion:**

The changes in the immune characteristics of CD8^+^ T cells may play a certain role in breaking down immune tolerance in patients with postpartum hepatitis flare, and the indexes related to activating and killing functions may help to indicate the population with hepatitis flare after childbirth.

## Background

Mother-to-child transmission was previously a major transmission route of hepatitis B virus (HBV) infection in China, accounting for about 40% of chronic infections (1, 2). To block mother-to-infant transmission, it is recommended in the Chinese and international guidelines that pregnant women with hepatitis B virus e antigen (HBeAg)-positive chronic HBV (CHB) infection (in the immune tolerance stage) can be treated with nucleoside analogue (NA) intervention combined with conventional immune blocking methods, which can substantially improve the success rate of blocking in the second and third trimesters of pregnancy (2–5). However, new clinical problems are emerging as more patients receive NA intervention in the third trimester of pregnancy; 25–45% patients will suffer postpartum hepatitis flare after drug withdrawal (6–8). The clinical features and the changes in the immune functions of such patients remain unknown at present. Therefore, the clinical features of patients with and without postpartum hepatitis flare were analyzed, and the differences in immune functions were preliminarily explored.

## Methods

### Study Population

The women with CHB infection who were pregnant (less than 8 weeks of gestation) and prepared for pregnancy, visited Beijing You’an Hospital, Capital Medical University from July 2017 to July 2018, and agreed on NA intervention in the third trimester of pregnancy to prevent mother-to-child transmission were enrolled. The inclusion criteria were as follows: 1) patients aged 18–40 years old, 2) those with positive hepatitis B virus surface antigens (HBsAgs) for more than 6 months, 3) those with positive HBeAgs, 4) those with an HBV DNA level ≥ 2 × 10^6^ IU/mL, 5) those with at least two normal test results of alanine transaminase (ALT) within one year before pregnancy, and 6) those without a history of antiviral treatment. The exclusion criteria involved 1) patients with infections of hepatitis A, hepatitis C, human immunodeficiency virus (HIV), or other viruses; 2) those with other chronic liver diseases (autoimmune liver disease, alcoholic liver disease, fatty liver, *etc.*); or 3) those with liver cirrhosis, liver cancer, or obstetrical disease (*e.g.*, intrahepatic cholestasis of pregnancy). All enrolled subjects signed the informed consent, and this study was reviewed by the Ethics Committee of Beijing You An Hospital.

### Study Methods

In this retrospective study, the aforementioned patients were followed up at baseline (in the second trimester of pregnancy, without antiviral treatment); before childbirth (at the 35^th^–38^th^ w of pregnancy); and after childbirth (at 6–12 w after delivery). HBV DNA, HBV serological markers, and hepatic and renal functions were detected at each follow-up. Postpartum hepatitis flare was defined as ALT ≥ 2 ULN (ULN = 40 U/L) during follow-up at 6–12 w after delivery and ALT ≥ 2 ULN in retest within 1 w.

#### Detection of Clinical Indexes

HBV DNA was examined using a COBAS TaqMan fluorescence quantitative polymerase chain reaction (PCR) system (Roche Diagnostics GmbH, Germany), with a lower limit of detection of 20 IU/mL. HBV serological markers were measured by Elecsys (Roche Diagnostics GmbH, Germany) (lower limit of detection of HBsAg: 0.05 IU/mL and lower limit of detection of HBeAg: 1 COI). Moreover, an OLYMPUS-AU5400 biochemical analyzer was applied to detect the hepatic and renal functions as well as other biochemical indexes.

#### Collection of Peripheral Blood Mononuclear Cells (PBMCs)

Peripheral venous blood was collected into 5 mL tubes containing anticoagulant EDTA from all subjects before and after childbirth. Then PBMCs (5 × 10^6^) were separated from each sample within 4 h after collection, cryopreserved in the cryopreservation container of a refrigerator at –80°C for 24 h, and transferred into a –130°C refrigerator for long-term storage.

#### Surface Staining *via* Flow Cytometry

The thawed cells were transferred into 37°C centrifuge tubes containing an RPMI-1640 medium, resuspended with a PBS buffer, and stained by means of such specific surface antibodies as CD3 APC-cy7, CD8 BV510, CD4 FITC, CCR7 Percp, CD45RA PE, CD38 PE-cy7, HLA-DR BV421. Later, the cells were washed and the expressions of markers were determined using a flow cytometer (BD Biosciences, San Jose, CA, USA). Flow cytometry Comp-Beads kits (BD Biosciences, San Jose, CA, USA) were used for compensation.

#### Intracellular Staining

The PBMCs (1×10^7^ cells/mL), CD3 (1 µg/mL), and CD28 (1 µg/mL) were added with GolgiStop and CD107a-FITC at the same time and then incubated at 37°C for 4 h. Next, the cells were collected, washed, and labeled with specific surface antibodies. Subsequently, the cells were collected, washed and fixed, followed by the rupture of membranes and the addition of interleukin-2 (IL-2) BV421, interferon gamma (IFN-γ) PE-Cy7, Perforin AF 647, and PE-GranB antibodies for incubation. Finally, the expressions of markers were measured by the flow cytometer.

### Statistical Analysis

The data were expressed as mean ± standard deviation. The data of flow cytometry were analyzed using FlowJo software (Version 10, Tree Star Inc., Ashland, OR, USA), and GraphPad Prism 6.02 (GraphPad software, San Diego, CA, USA) was used for other statistical analyses. The intergroup differences in *p* values with statistical significance were verified by means of a Mann-Whitney test and a one-way analysis of variance. Spearman’s rank correlation test was conducted to assess the statistical correlation between variables. All the tests were two-tailed, and *p* < 0.05 indicated a significant difference.

## Results

### General Conditions of the Patients

A total of 25 patients with an average age of 28.2 ± 4.3 years old were included in this study, and they started to take tenofovir disoproxil fumarate (TDF) in the second and third trimesters (24–28 w) of pregnancy and stopped the drug at 3 ± 2 w after childbirth. Then the patients were assigned to a hepatitis group (Group 1, *n* = 15) and nonhepatitis group (Group 2, *n* = 10), based on the ALT level within 12 w after childbirth. No statistical differences in the age, baseline ALT level, and baseline HBV DNA level were observed when comparing the two groups of patients. The baseline, antepartum, and postpartum clinical features of the patients appear in **Table 1**.

**Table 1:** Characteristics of all subjects with and without postpartum hepatic flares.

Characteristic	Group 1 Postpartum flares (n=15)	Group 2 No postpartum flares (n=10)	*P*-value
**Age (years)**	26.8 ± 2.8	30.0 ± 5.4	0.182
**ALT (U/L)**			
Baseline	26.0 ± 9.6	20.8 ± 7.7	0.510
Gestational 35-38W	20.9 ± 8.2	18.6 ± 8.0	0.376
Postpartum 6–12W	**113.9 ± 40.3**	**32.0 ± 9.8**	**<0.0001**
**HBV DNA (log_10_ IU/ml)**			
Baseline	7.9 ± 0.7	7.9 ± 0.6	0.713
Gestational 35-38W	4.8 ± 0.8	4.6 ± 0.5	0.999
Postpartum 6–12W	4.7 ± 1.7	5.1 ± 2.3	0.403
**HBsAg (log_10_ IU/ml)**			
Baseline	4.3 ± 0.5	4.5 ± 0.5	0.249
Gestational 35-38W	4.2 ± 0.4	4.4 ± 0.5	0.077
Postpartum 6–12W	**4.1 ± 0.5**	**4.4 ± 0.5**	**0.022**
**HBeAg (log_10_ COI)**			
Baseline	3.3 ± 0.1	3.2 ± 0.1	0.113
Gestational 35-38W	3.3 ± 0.1	3.2 ± 0.1	0.673
Postpartum 6–12W	3.0 ± 0.4	3.2 ± 0.2	0.099

The bold values means the difference is statistically significant.

### Comparisons of Clinical Features Between the Two Groups

**Figure 1:**
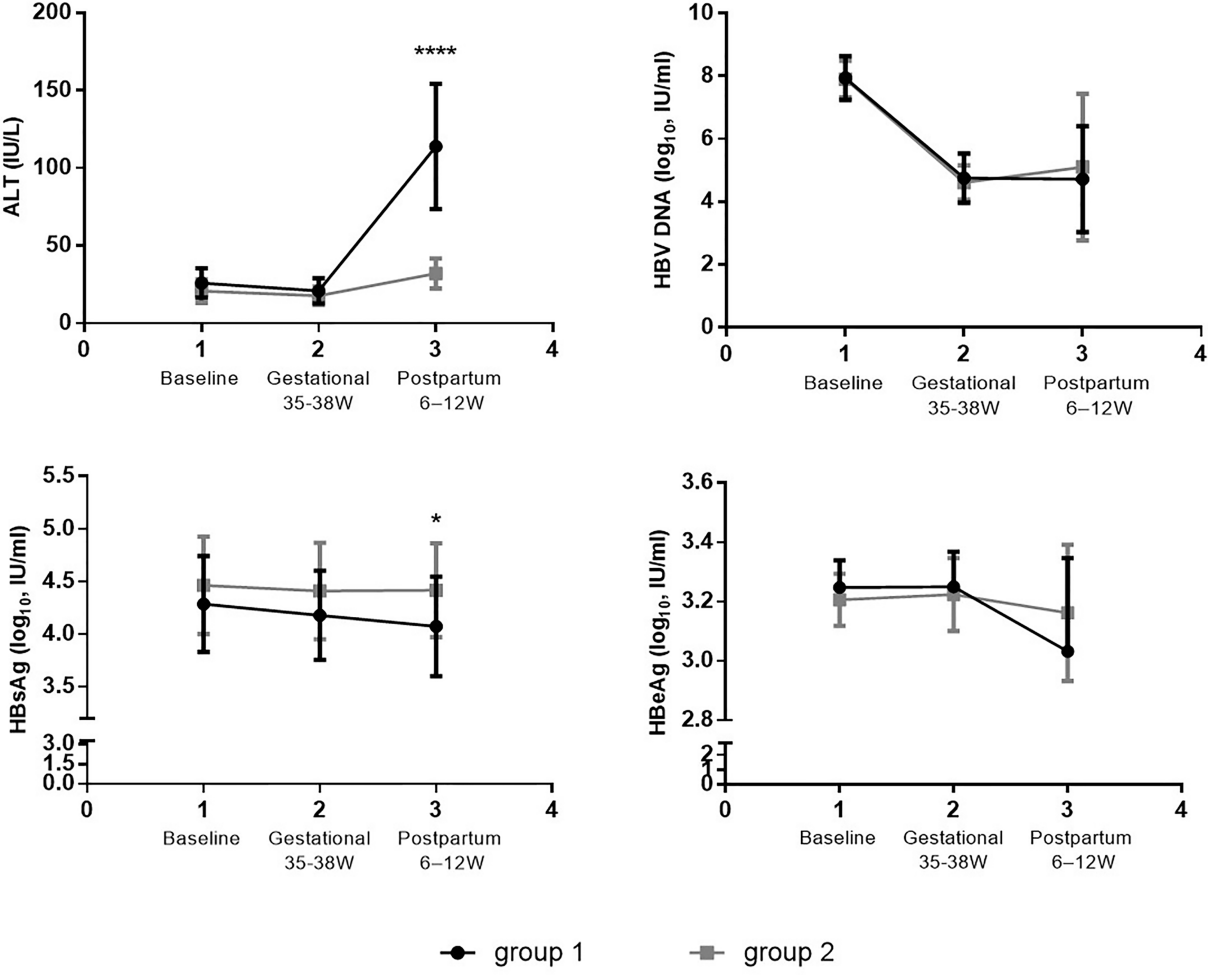
clinical features between the two groups. Compared the difference of the levels of ALT, HBV DNA, HBsAg and HBeAg at each follow-up point between the group 1 (n=15) and group 2 (n=10). *p < 0.05, ****p < 0.0001.

Longitudinal comparisons within groups and horizontal comparisons between groups were adopted for the clinical features of the two groups of patients at baseline, before childbirth, and after childbirth. As for the changes within groups, the ALT level rose markedly after childbirth compared with that at baseline in Group 1 (*p* < 0.0001). It also increased in Group 2 (*p* = 0.006), but the extent was below the standard of a hepatitis flare. The HBV DNA level declined evidently (by up to 3log) before and after childbirth in both groups by contrast with that at baseline (*p* < 0.0001). The HBsAg and HBeAg levels lowered in Group 1 after childbirth by comparison with those at baseline, while the HBeAg level decreased more distinctly, with a statistical difference (*p* = 0.004). However, there were no apparent changes in the HBsAg and HBeAg levels at the three follow-up points in Group 2. Moreover, the ALT and HBsAg levels displayed obvious changes after childbirth in both groups. Specifically, Group 1 had a prominently higher ALT level and a lower HBsAg level than Group 2, showing statistically significant differences (*p* < 0.0001) (**Figure 1**).

### Activation of CD8^+^ T Cells and Their Subsets

**Figure 2:**
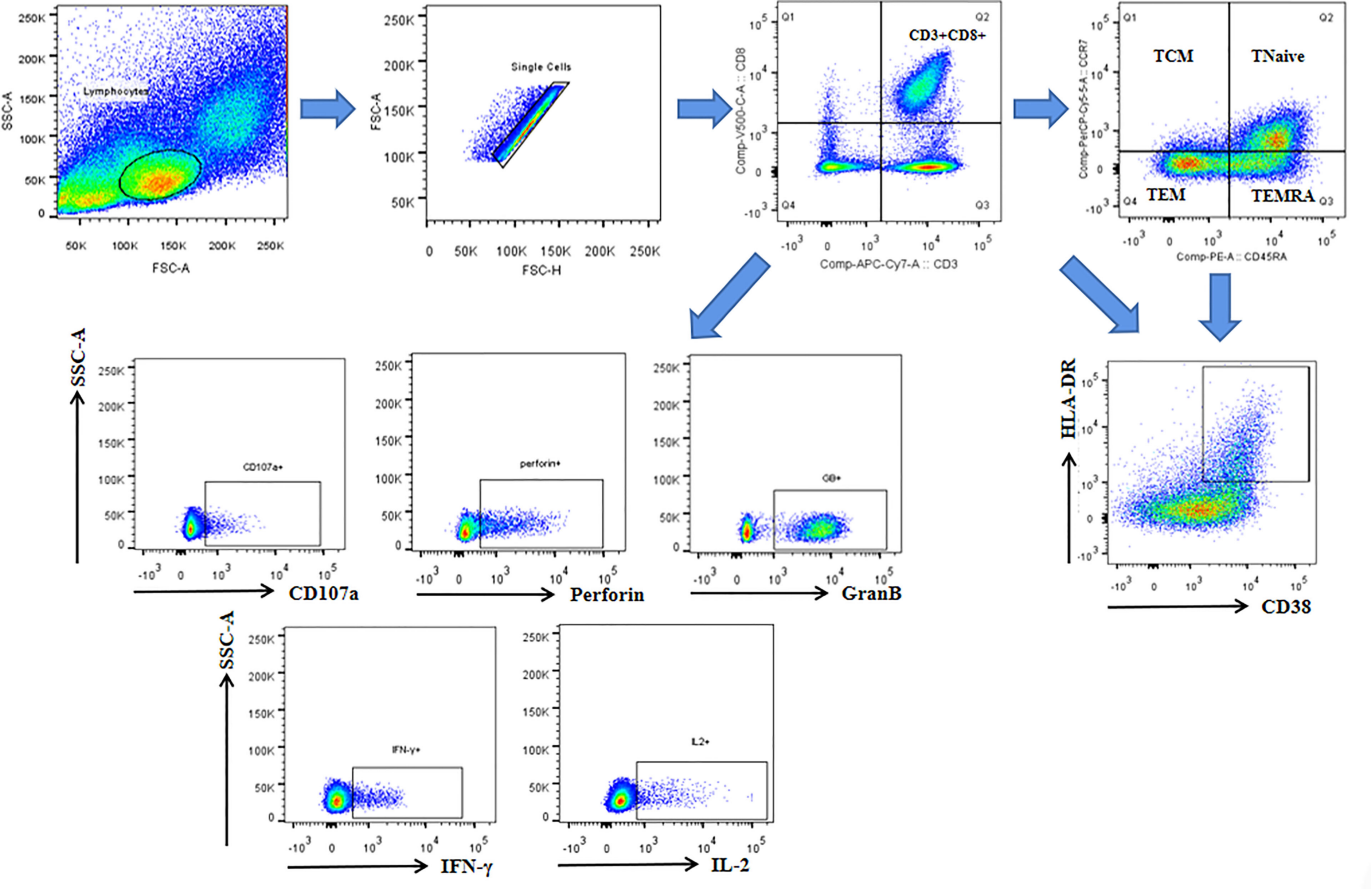
The gating strategy of CD8^+^ T cells populations and other markers. Lymphocytes and single cells were gated first. Then CD3^+^CD8^+^cells were gated. Central memory (TCM: CD45RA^-^CCR7^+^), Tnaive (CD45RA^+^CCR7^+^), effector memory (TEM: CD45RA^-^CCR7^-^), and terminally differentiated effector (TEMRA: CD45RA^+^ CCR7^-^) subsets were gated based on gated CD8^+^ cells. CD38 and HLA-DR were gated based on gated CD8^+^ cells and each subsets. CD107a, perforin, GranB, IFN-γ and IL-2 were gated based on gated CD8^+^ cells.

**Figure 3:**
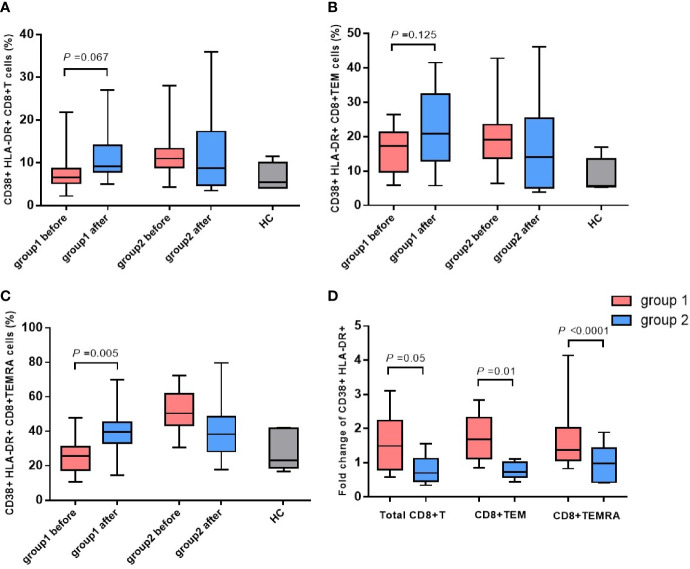
The changes in CD38 and HLA-DR expressed CD8^+^ T cells and phenotypic subsets. Expression of CD38 and HLA-DR on CD8^+^ T cells **(A)**, TEM subsets **(B)** and TEMRA subsets **(C)** before and after childbirth between the group 1 and group 2. Fold change of activation of CD8^+^ T cells, TEM subsets, and TEMRA subsets after childbirth between the group 1 and group 2 **(D)**.

The antepartum and postpartum activations of CD8^+^ T cells and their subsets were compared between the two groups to analyze whether hepatitis flare is associated with immune changes. The gating strategy of CD8^+^ T cells populations and other markers was shown in **Figure 2**. The results indicated that the frequency of CD38^+^ HLA-DR^+^ CD8^+^ T cells exhibited an increasing trend after childbirth in Group 1, suggesting enhanced postpartum activation, but such a phenomenon was not observed in Group 2. Besides, there was no statistically significant difference between the two groups (*p* = 0.067, **Figure 3A**). Next, the cell subsets playing key roles in anti-infectious immunity, namely, CD8^+^ effector memory T cells (CD45RA^-^CCR7^-^, TEM) and effector T cells (CD45RA^+^CCR7^-^, TEMRA), were further analyzed. The activation of TEM and TEMRA subsets was strengthened after childbirth compared with that before childbirth in Group 1, and in particular, there was a statistically significant difference in the enhancement of TEMRA subset activation after childbirth (*p* = 0.005, **Figures 3B, C**). However, Group 2 displayed no such phenomena. To eliminate confounding factors and observe the activation of CD8^+^ T cells more intuitively, TEM subsets and TEMRA subsets before and after childbirth, along with fold change (postpartum numerical value/antepartum numerical value) were used to evaluate the change degree. According to the results, the fold changes of activation of CD8^+^ T cells, TEM subsets, and TEMRA subsets after childbirth were markedly higher in Group 1 than in Group 2 (*p* = 0.05, *p* = 0.01, *p* < 0.0001, **Figure 3D**), implying that postpartum hepatitis flare in immune-tolerant HBV patients may be correlated with changes in immune function.

### Detection Results of Killing Function and Cytokines of CD8^+^ T Cells

**Figure 4:**
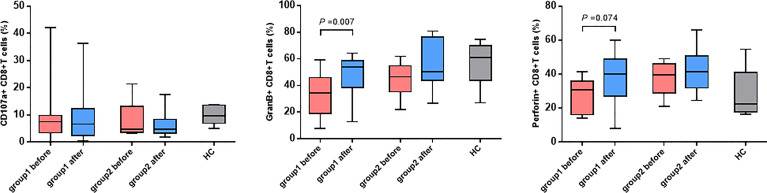
The changes in CD107a, GranB and perforin expressed CD8^+^ T cells. Expression of CD107a, perforin and GranB on CD8^+^ T cells before and after childbirth between the group 1 and group 2.

**Figure 5:**
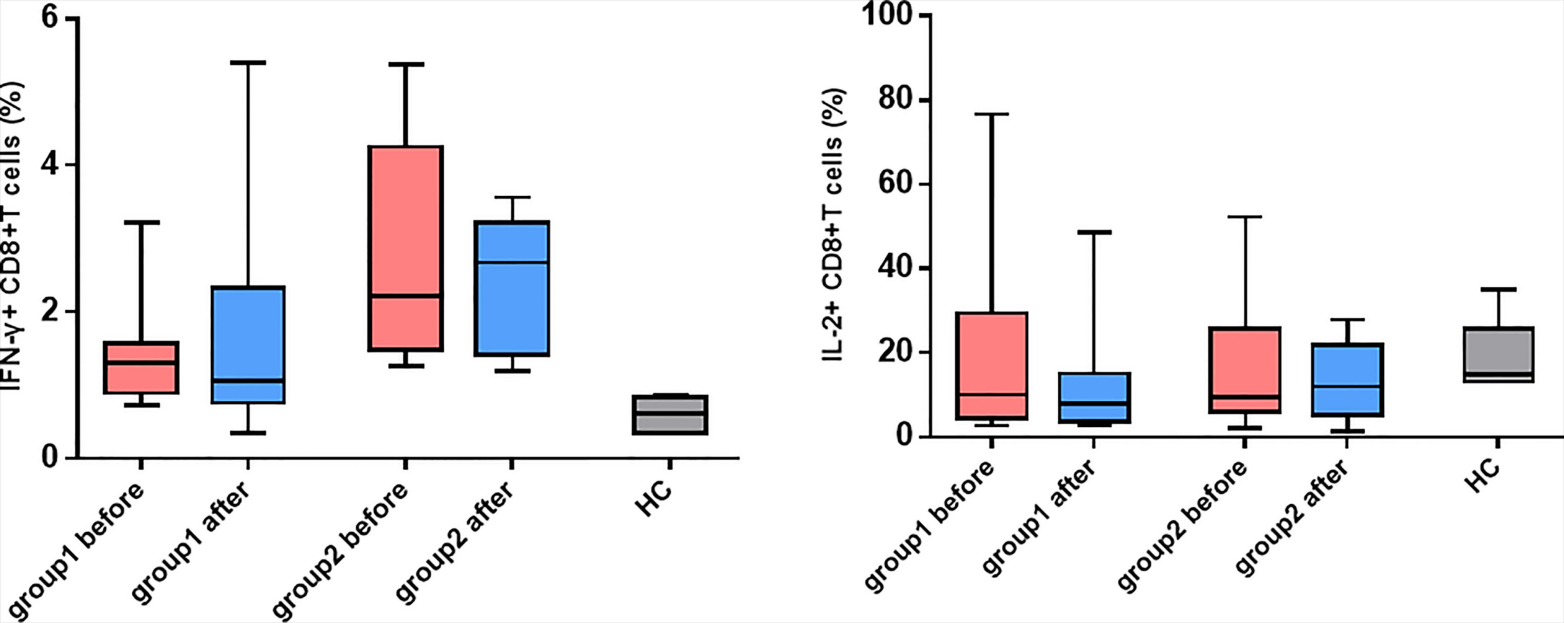
|The changes in IFN-γ and IL-2 expressed CD8^+^ T cells. Expression of IFN-γ and IL-2 on CD8^+^ T cells before and after childbirth between the group 1 and group 2.

In light of the significant difference in CD8^+^ T cell activation between the two groups of patients, whether the killing function and cytokine secretion of CD8^+^ T cells affected hepatitis flare was further analyzed. The antepartum and postpartum expressions of CD107a, perforin, and granzyme B in the two groups of patients were measured using the flow cytometer. It was revealed that the expression of granzyme B in CD8^+^ T cells increased notably after childbirth in Group 1, and the difference was statistically significant (*p* = 0.008). In addition, the perforin expression increased, but it showed no statistically significant difference (*p* = 0.074). Moreover, the CD107a expression was unchanged (**Figure 4**). By contrast, the increases in CD107a, perforin, and granzyme B expressions were not observed after childbirth in Group 2. Furthermore, the antepartum and postpartum IFN-γ and IL-2 levels in the two groups were determined, and it was discovered that there were no apparent changes in the levels of the two cytokines between the two groups (**Figure 5**).

## Discussion

In recent years, NA intervention, combined with conventional immune blocking methods, has been used for more and more immune-tolerant pregnant women in the second and third trimesters to improve the success rate of blocking, which has substantially increased the success rate of blocking in pregnant women with high HBV load and neonates (2, 4, 5). Nevertheless, multiple researchers have reported that 25–45% patients have ALT elevation and hepatitis flare after childbirth (6, 8), and it was noted in the research that postpartum hepatitis flare is good timing for antiviral treatment (9). The reason the extent of postpartum ALT elevation differs greatly among patients with HBV immune tolerance undergoing NA intervention, the changes in related immunological indexes, and whether these changes can predict hepatitis flare are rarely reported.

In terms of the features of postpartum hepatitis flare, unlike those of an ordinary CHB infection, ALT elevation may occur at a relatively low DNA level before or immediately after drug withdrawal, whereas hepatitis flare emerges in CHB generally at a high HBV DNA level (6, 10, 11). This study showed that obvious HBV DNA decline and ALT elevation were detected in Groups 1 and 2 after childbirth, with statistical differences with those individuals at baseline, but the ALT elevation in Group 2 did not reach the standard of hepatitis flare. Besides, the HBeAg level in Group 1 was also reduced clearly after childbirth in contrast with the level at baseline, and the difference was statistically significant (*p* = 0.004), but this phenomenon was not noted in Group 2. Additionally, Group 1 exhibited an overtly higher overall ALT level and an obviously lower HBsAg level after childbirth than Group 2, showing statistically significant differences. However, these changes were representations rather than causes of postpartum hepatitis flare; the specific immunological changes remain unclear and need to be more deeply explored.

Regarding the pathogenesis of CHB, it is generally argued that CD8^+^ T cell immune responses exert crucial effects on HBV clearance, but the dysfunction or failure of T cells during long-term viral infection can lead to immune tolerance (12). TEM and TEMRA are important CD8^+^ T cell subsets, and the former is probably a crucial type of effector cell for early elimination after viral infection. It was reported that viruses are gradually removed as the TEM level becomes more elevated in the case of acute hepatitis B, influenza, and other adult viral diseases (13). This phenomenon suggests that the number and function of TEM subsets are critical players in eliminating viruses and preventing chronicity of disease. According to the study of Hess et al. on HIV infection, the fully differentiated TEMRA subsets can express more perforin than TEM subsets, so they can control viral infection more effectively (14). Ma et al. detected CD8^+^ T cell subsets in different HBV infection states (spontaneous clearance of HBsAg and HBeAg, CHB, *etc.*), finding that the obvious increase in TEMRA subsets could serve as an important mechanism for HBV clearance (including the disappearance of HBV DNA and HBV-related antigens) (15). Similar phenomena were observed in the present study as well. The activation of three cell sets (CD8^+^ T cells, TEMRA subsets, and TEM subsets) was more prominently enhanced after childbirth in Group 1 than in Group 2. In particular, there was a significant difference among the TEMRA subsets (*p* = 0.005). Additionally, the fold changes of activation of the three cell sets after childbirth were remarkably higher in Group 1 than in Group 2; TEMRA subsets displayed the greatest difference, implying that TEMRA subsets may play vital roles in breaking down immune tolerance and inducing hepatitis flare.

CD8^+^ T cells can exert cytotoxic effects by virtue of the cell-killing effect mediated by perforin and granzyme and the non-cell-killing effect mediated by secreted cytokines such as IFN-γ, TNF-α, and IL-2 (16). It was also observed in this study that the frequency of CD8^+^ T cells secreting perforin and granzyme B clearly increased more after childbirth in Group 1 than in Group 2, and there was a statistical difference (*p* = 0.008), similar to the findings of Hess et al. (HIV) and Duan et al. Duan et al. discovered that a larger number of CD8^+^ T cells secreted perforin and granzyme B in CHB patients who had negative HBeAg after TDF treatment (17). There were no obvious changes in cytokines IFN-γ and IL-2 after childbirth in either group. In a study on similar populations to those in this study, the postpartum IL-2 level had no variation, but IFN-γ expression was higher in patients with hepatitis flare than in patients without hepatitis flare (18). The different results between the two studies could be attributed to the differences in time of drug (NA) withdrawal and postpartum follow-up points of enrolled patients. The test results could have been influenced by the varying time points of blood collection, given that corticosteroids withdraw rapidly after childbirth and could be associated with the small sample size in the present study. The relatively small sample size of this study may limit the power to obtain the desired effect, we will expand the sample size and conduct more comprehensive experimental design in future research, such as analysis of CD4^+^ T cell immunity.

In conclusion, the clinical features of postpartum hepatitis flare in patients subjected to NA intervention include a remarkable elevation of the ALT level on the basis of a relatively low HBV DNA level, usually accompanied by a prominent decline in HBsAg and HBeAg levels. Patients with hepatitis flare manifest evident activation of CD8^+^ T cells, and increased frequency of TEMRA subsets and expression levels of perforin and granzyme B may perform crucial functions in breaking down immune tolerance and causing hepatitis flare.

## Data Availability Statement

The raw data supporting the conclusions of this article will be made available by the authors, without undue reservation.

## Ethics Statement

The studies involving human participants were reviewed and approved by Beijing Youan Hospital Research Ethics Committee. The patients/participants provided their written informed consent to participate in this study.

## Author Contributions

AS, YL, and XC conceived and designed the experiments and study. ZC, JL, and SR collected the sample information, contributed to reagents and materials. YL, YZ, XL, and HL performed the experiments and analyzed the data. AS performed the analysis and wrote the manuscript with assistance from SZ, ZH, LM, and XC. All authors read and approved the final manuscript.

## Funding

This work was supported by Capital Health Research and Development Projects (2020-1-2181), the Capital Clinical Diagnostic Techniques and Translational Application Projects (Z211100002921059), the Beijing Municipal Administration of Hospitals’ Youth Program (QML20211702), the Beijing Municipal Administration of Hospitals Clinical medicine Development of special funding support (ZYLX202125), National Science and Technology Key Project (2017ZX10302201-004, 2017ZX10202203-006). Beijing Natural Science Foundation (No. 7222093), Chinese National Natural Science Foundation (81900537).

## Conflict of Interest

The reviewer YX has declared a shared parent affiliation with the authors to the handling editor at the time of review.

The remaining authors declare that the research was conducted in the absence of any commercial or financial relationships that could be construed as a potential conflict of interest.

## Publisher’s Note

All claims expressed in this article are solely those of the authors and do not necessarily represent those of their affiliated organizations, or those of the publisher, the editors and the reviewers. Any product that may be evaluated in this article, or claim that may be made by its manufacturer, is not guaranteed or endorsed by the publisher.
